# Ac-225 radiochemistry through the lens of [^225^Ac]Ac-DOTA-TATE

**DOI:** 10.1186/s41181-025-00332-z

**Published:** 2025-02-20

**Authors:** Eline L. Hooijman, Jan R. de Jong, Carolline M. Ntihabose, Frank Bruchertseifer, Alfred Morgenstern, Yann Seimbille, Tessa Brabander, Stijn L. W. Koolen, Erik de Blois

**Affiliations:** 1 Department of Radiology and Nuclear Medicine, Erasmus MC, 3015 CN Rotterdam, The Netherlands; 2 Department of Hospital Pharmacy, Erasmus MC, 3015 CN Rotterdam, The Netherlands; 3 Joint Research Centre, European Commission, 76344 Karlsruhe, Germany; 4 Division of Life Sciences, TRIUMF, Vancouver, BC V6T 2A3 Canada; 5 Department of Medical Oncology, Erasmus MC Cancer Institute, 3015 CN Rotterdam, The Netherlands

**Keywords:** Targeted alpha therapy (TAT), Ac-225/Fr-221, Quality control, Radiopharmaceutical implementation, Radiochemical yield, Radiochemical purity

## Abstract

**Background:**

Targeted alpha therapy with Ac-225 showed to be effective in treating metastatic cancers. However, the complex decay chain requires optimized radiolabeling and quality control. This study aims to determine critical parameters and establish optimal labeling and accurate measuring techniques for radiochemical yield and purity with DOTA-TATE as a model molecule. Ac-225 sources were analyzed for metals (ΣFe, Zn, Cu) and quantified by UPLC. Optimization of radiolabeling kinetics for clinical conditions was performed in regards to temperature (20–90 °C), heating time (5–60 min), pH (2.5–10, with/without excess of metal ions), buffers, quenchers, volume (0.1–10 mL) and molar activity (90–540 kBq/nmol). The quality control was investigated using radio-TLC/HPLC by changing gradient to evaluate peak separation, radiolysed peptide and impurity separation.

**Results:**

Metal ingrowth was observed in Ac-225 stocks (*n* = 3), (time of arrival: 17.9, 36.8 and 101.4 nmol per 10 MBq). Optimal radiochemical yields were achieved with > 80 °C (20 min) at pH 8.5 (15 mM TRIS) up to 270 kBq. Labeling at a high pH showed a higher RCY, even in presence of an excess of metals. High stability (RCP > 90%) was achieved after addition of quenchers (cysteine, methionine, ascorbate, histidine, or gentisic acid (35 mM)) up to 24 h. For optimal determination of the radiochemical purity (indirect HPLC) fifty fractions are required.

**Conclusion:**

The quality of Ac-225 labeled DOTA-radiopharmaceuticals is highly dependent on the pH and stabilization (buffer/quencher). Within this research it is demonstrated that optimized quality control methods and accurate measurement of the radiolabeling kinetics are crucial to ensure safe implementation for patient treatment.

## Introduction

Due to the tumor-specific targeting characteristics, in recent years, targeted radionuclide therapy (TRT) has increasingly gained interest in the field of nuclear medicine, specifically for the treatment of metastatic cancer(s). TRT leverages the unique properties of the radionuclides to deliver cytotoxic radiation directly to the cancer cells, minimizing the damage to the surrounding and off-target healthy tissues. This approach has demonstrated encouraging outcomes for neuroendocrine tumors (NETs) (Han et al. 2023) and (metastatic) prostate cancer (PCa) (Ling et al. 2022). The TRT therapies have the potential as an effective treatment modality, especially for cancers that are resistant to conventional care or if surgical options are limited (Harris and Zhernosekov 2022; Seo 2019; Kratochwil et al. 2019).

One of the developments in TRT is the use of an alpha emitter such as actinium-225 (Ac-225) for radiolabeling with various biological vectors (Kratochwil et al. 2018; Feuerecker et al. 2021; Shi 2022; Hatcher-Lamarre et al. 2021; Kim and Brechbiel 2012), instead of the conventional beta emitters such as lutetium (Lu-177) (Armstrong 2024; Becx, et al. 2022). Due to the alpha radiation emitted by Ac-225, and the subsequent high linear energy transfer (LET), a potent local dose and cytotoxic effect on the target can be expected. As the potential of Ac-225 has grown, so has the interest in optimizing the quality control (QC) methods and radiolabeling process for Ac-225. Currently, there are no European Pharmacopoeia (Ph. Eur.) Ac-225 based radiopharmaceutical monographs available, but merely guidelines such as the Guideline on Current Good Radiopharmacy Practice (cGRPP) and European Association of Nuclear Medicine (EANM) recommendations (Gillings et al. 2021; Gillings et al. 2022; Gillings et al. 2020). Furthermore, despite the promising applications of Ac-225 in TAT, the fundamental reaction kinetics for the DOTA-conjugated radiopharmaceuticals are not systematically investigated. Ac-225 exhibits behaviors that differ from other radionuclides used for TRT such as Lu-177 (therapy) and gallium-68 (Ga-68, diagnostic). Due to the complex decay chain of Ac-225 and its radiotoxic daughter isotopes, the QC of these radiopharmaceuticals is critical and accurate measurement of the radiochemical yield (RCY) and radiochemical purity (RCP) is required. Additionally, alpha-particle decay causes recoil and the detachment from the chelator.

Therefore, detection of alpha emission and potential damage of the radiopharmaceutical, creates a new type of challenge for the implementation of Ac-225 labeled DOTA-radiopharmaceuticals (Hooijman et al. 2024).

A widely recognized golden standard as a pharmaceutical within the field of TRT is DOTA-TATE, a small peptide (1436 g/mol) that exhibits high affinity for somatostatin receptors (SSTRs), commonly overexpressed in NETs (Becx et al. 2022). DOTA-TATE has been extensively used and is known for the high stability (Blois et al. 2014; Breeman et al. 2016). However, when DOTA-conjugated radiopharmaceuticals are labeled with Ac-225, the reaction conditions deviates (Hooijman et al. 2024; Hooijman et al. 2021) and therefore, detailed information on the radiochemistry of Ac-225 is essential to further understand the reaction kinetics to optimize the process for a safe and effective use.

Due to the challenges of Ac-225 in regards to the detection of the alpha emission, and due to the high LET energy, accurate measurement of the RCY and RCP is found to be challenging. Therefore, this research aims to identify the critical parameters for optimal radiolabeling and to establish accurate measurement techniques for the RCY and RCP for Ac-225-labeled DOTA-conjugated radiopharmaceuticals, using DOTA-TATE as a model.

## Materials and methods

### Materials and chemicals

All stock solutions were prepared in quartz-coated vials (Curium, Petten, The Netherlands) and the purchased chemicals were dissolved in MilliQ water (HiPerSolv Chromanorm, VWR Chemicals, Amsterdam, The Netherlands).

The buffer and quenching solutions were prepared at a concentration of 100 mM unless stated otherwise. The 1 M HCl (from 37% HCl) and 10 M NaOH (Sigma Aldrich Chemicals BV, Zwijndrecht, The Netherlands) were used to set the pH of a TRIS-buffer at pH 9 (Merck chemicals BV, Amsterdam, The Netherlands) and ascorbate solution (VWR Chemicals BV, Amsterdam) to pH 5.5, respectively. The DOTA-TATE (Bachem AG, Bubendorf, Switzerland) was dissolved to a concentration of 0.6 nmol/µL with MilliQ. Directly after each labeling, diethylenetriaminepentaacetic acid (DTPA, 4 mg/mL) (Hospital pharmacy A15, Gorinchem, The Netherlands) was added to the labeling solution to terminate the reaction.

Radiolabelling preparations with Ac-225 were performed in a dedicated hotcell. The Ac-225 was diluted in 0.1 M HCl (Eckert & Ziegler, Berlin, Germany), and the radiolabeling was performed in either a MoBiTec vial in a heating block (Sarstedt, Darmstad, Germany) or for > 10 MBq labeling in v-shaped microwave vial in a dedicated microwave (Biotage Initiator + , Biotage, Uppsala, Sweden) (Hooijman et al. 2021).

### Metal quantification

The Th-229 based Ac-225 was provided by multiple vendors, and obtained via Van Overeem Nuclear B.V. (Breda, The Netherlands) and the Joint Research Centre, Karlsruhe (JRC, Karlsruhe). All sources were tested for metal content (Fe, Zn and Cu). To investigate the metal impurities, a molar activity of 200 kBq/20 nmol DOTA-TATE was labeled, see Table 1 for the reaction conditions. The reaction mixture was heated for 20 min at 90 °C and after cooling, an excess of DTPA was added. The samples were analyzed by UPLC (*n* = 3) according to the method described by de Blois et al. (Breeman et al. 2014).

**Table 1:** Labeling conditions for metal impurity quantification

Parameter of interest	Ac-225 activity (kBq)/ Peptide mass (nmol)	Concentration of buffer in labeling vial
Metal impurities (Fe, Zn, Cu)	10	15 mM TRIS (pH = 3.0), *n* = 3

### Radiolabeling

The starting point for the radiolabeling with Ac-225 was based upon the [^177^Lu]Lu-DOTA-TATE conditions (Table 2) (Blois et al. 2014). Subsequently, the conditions were optimized for Ac-225 using a molar activity of 90 kBq/nmol; the described molar activity is based on a final clinical dose of 12 MBq/192 µg (Blois et al. 2014). All concentrations are described as final concentrations; the total labeling volume was 100 µL, unless otherwise specified.

**Table 2:** Conditions for labeling optimization and QC of Ac-225 labeled DOTA-TATE

Parameter of interest	Ac-225 activity (kBq)/Peptide mass (nmol)	Final concentration in labeling vial
Heating time, *n* = 2	90	2 mM gentisic acid/8 mM ascorbate (Blois et al. 2014)
Heating temperature, *n* = 3		
Optimization HPLC-gradient, *n* = 2		
Optimization HPLC-fractionation, *n* = 2		
Radiolysis separation, *n* = 3	90	15 mM TRIS (pH = 8.5) (Hooijman et al. 2021), 15 mM Sodium Acetate (pH = 6.0) (Pretze et al. 2021; Mikolajczak et al. 2019), 35 mM Ascorbate (pH = 5.5) (Hooijman et al. 2021)

The labeling conditions used for optimization and the QC (radio-TLC/RCY, HPLC/RCP gradient, fractionation and radiolysis separation) are described in Table 2.

The influence of the pH during labeling, the used buffers, quenchers, volume of radiolabeling, molar activity and storage were investigated by monitoring the RCY and/or the RCP. An overview of the reaction conditions is summarized in Table 3.

**Table 3:** Optimization of labeling conditions

Parameter of interest	Ac-225 / Peptide mass (kBq /nmol)	Final concentration in labeling vial
pH, *n* = 3	90	15 mM TRIS (pH varying 10, 8.5, 7.0, 6.5, 4.0, 2.5)
Metal excess, *n* = 3	90	15 mM TRIS (pH varying 5.5 and 8.5) Spiked with 1x/10 × excess of Fe, Zn and Cu
Buffering, *n* = 2	90	15 mM/1 M TRIS (pH = 8.5) 15 mM/1 M Sodium acetate (pH = 6.0) 35 mM Ascorbate (pH = 5.5) (Pretze et al. 2021; Reissig et al. 2021; Kratochwil et al. 2021)
Quenchers, *n* = 1	90	15 mM TRIS (pH = 8.5) + 35 mM cysteine, methionine, ascorbate, histidine, gentisic acid*, nicotinamide, vanillin or 10% v/v EtOH
Volume, *n* = 3	90	35 mM Ascorbate (pH = 5.5) in end volume of 0.1, 0.5, 1 or 10 mL
Molar activity, *n* = 3	90, 180, 360, 540	15 mM TRIS (pH = 8.5) or 35 mM Ascorbate (pH = 5.5)
Storage after radiolabeling, *n* = 2	90	35 mM Ascorbate (pH = 5.5) Dry-ice/-20 °C storage

*Due to the pH of < 2 in the labeling solution after addition of gentisic acid, pH stabilization was performed with 52 mM TRIS

### Systems and settings

The determination and quantification of metal impurities in the Ac-225 stock was performed using a UPLC (Acquity H-class system including an Acquity UPLC HSS C18, 1.8 µm 2.1 × 50 mm column). The system was equipped with a sample manager, and a PDA 2998 UV detector controlled by Empower 3 software (Waters, Ette-Leur, and the Netherlands). A validated UPLC-method was used, as described by de Blois et al. (2014) (Breeman et al. 2014).

The QC measurements are based upon the Ac-225/Fr-221 equilibrium, using the 218 keV gamma emission of Fr-221 (± 11% yield). After 6 half-lives of Fr-221 (T½ = 4.8 min, 30 min), a secular equilibrium with Ac-225 is achieved (Hooijman et al. 2024).

Quantification of the activity was performed with a dose calibrator (VIK-202 and VDC 404 Comecer, Castel Bolognese, Italy); the RCY was measured by radio-TLC (bSCAN, Brightspec, Zelik, Belgium). The RCP was based on indirect measurement of the HPLC-analyses (Alliance 2695XE HPLC, Waters Chromatography B.V., Etten-Leur, The Netherlands) whereby the fractions are collected with a automated Wizard III fraction collector (Waters Chromatography B.V., Etten-Leur, The Netherlands) (Gillings et al. 2021; Gillings et al. 2020; Hooijman et al. 2024). The collected fractions (10, 25, 50 and 100 fractions), were measured using a gamma counter (automated gamma counter 2480 Wizard-2, Perkin Elmer, Waltham, MA, USA). The used HPLC quality control was validated according the EANM guidelines, the parameters are described in Table 4.

**Table 4:** Parameters required for the validation of HPLC-methods

Parameter	Description	Norm	Result
Recovery	Percentage of activity that can be measured after injection	100% ± 10	105.3 ± 3.4
Carry-over	Percentage of activity that remains from the previous analysis	< 1%	0.5 ± 0.3
LOD/LOQ	Value at which the detection (LOD) and quantification (LOQ) is possible	3 × SD = LOD 10 × SD = LOQ	Indirect measurement, gammacounter (Fr-221); 78 cpm (LOD), 260 cpm (LOQ)
Specificity	Baseline separation of the impurity (Ac-225/[^225^Ac]Ac-DTPA, radiolysed) peak and labeled peak Deviation of the retention time in relation to non-active standard	Resolution factor > 1,5 Retention time deviation < 1%	> 1.5 < 1%
Precision (repeatability)	Deviation of the measurements when repeated (retention factor (rf))	< 5%	< 1%
Linearity	Linear range of measurements	R^2^ > 0.98	R^2^ > 0.99
Accuracy	Measuring the impurities after intentionally spiking with 10% [^225^Ac]Ac-DTPA	9–11%	10% ± 0.5

For the RCY analysis, 1 × 10 cm SG-TLC strips are used as a stationary phase. Sodium citrate (0.1/0.5 M) was used as a mobile phase.

For HPLC-analysis, a mobile phase containing 0.1% Trifluoroacetic acid (TFA) (A) and methanol (B) was used. The HPLC samples for the QC were prepared by taking an aliquot from the labeling mixture, which was diluted in 25% EtOH (LC–MS grade Chromasolv Ethanol, Sigma Aldrich, Zwijndrecht, The Netherlands) to a concentration of 10 kBq/100 µL into a 300 µL polypropylene vial (Waters, Etten-Leur, The Netherlands). Different HPLC-gradients (Methods A-C) were tested to investigate and optimize peak separation. For each of the methods, the gradients are listed in Table 5 (mobile phases: 0.1% TFA (A)/methanol (B)).

**Table 5:** Overview of used HPLC-gradients and column

	Gradient	Column
A	0–2 min 100% A (flow 1 mL/min), 2–3 min 55% B (flow 0.5 mL/min), 3–20 min 60% B (flow 0.5 mL/min), 20–20.01 min 100% B (flow 1 mL/min), 20.01- 25 min 100% A (flow 1 mL/min), 25.01- 30 min 100% A (flow 1 mL/min)	Symmetry C-18 (4.6 × 250 mm, 5 μm, Waters)
B	0–15 min 85% A, 15–20 min 100% B, 20–25 min 85% A (flow 1 mL/min)	
C	1–20 min 85% A, 20–25 min 100% B, 25–30 min 85% A (flow 1 mL/min)	

The optimal fraction number was determined using method A, 10, 25, 50 or 100 tubes (borosilicate glass tube (Pyrex 12 × 7.5 mm)) were collected within the 30 min gradient, while keeping other conditions constant.

As health physics regulations only allow low amounts of activity for HPLC-analysis (~ 10 kBq), the validation of the recovery and carry-over is challenging. This is complicated as the measurements are close to the LOD (Aalbersberg et al. 2022). Both parameters were validated using a standard labeling (Sect. ”Systems and Settings”), whereby the waste after HPLC-injection was collected in 50 mL Falcon tubes (Corning, New York, United States) with addition of 1 mL 0.1% human serum albumin (HSA) (Sigma Aldrich, Darmstadt, Germany), to prevent sticking.

**Fig. 1:**
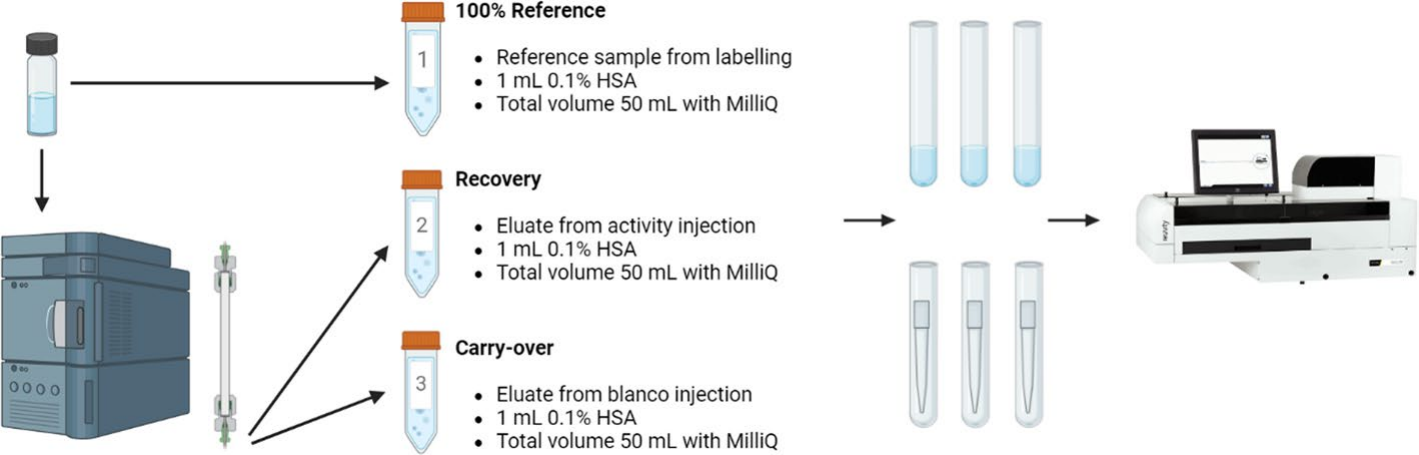
Procedure for measuring the recovery and carry-over

In the first tube, 100% of the injected amount is added into 50 mL MilliQ/1 mL 0.1% HSA. Secondly, the HPLC-eluate was collected in tube 2, and in tube 3, the non-active injection eluate is collected. All tubes were filled to 50 mL, to correct for dilution. After vortexing, 10 times 1 mL was pipetted into a plastic 10 mL reaction tube (Sarstedt, 8.5 mL, 75 × 15.7 mm), additionally, each pipet tip was measured (Fig. 1). The recovery and carry-over were calculated as described (see formula 1).

1 $$Recovery/Carry-over=\frac{Activity\; tube\; of\; interest}{Activity\; reference\; tube}*100\%$$

Equation to calculate recovery and carry-over.

The optimization of the recovery was further investigated using a RP-18 column (LiChrospher® 100 RP-18 endcapped (4 × 250 mm, 5 µm), Merck, Darmstadt, Germany) was implemented in comparison with the Symmetry C-18 column (4.6 × 250 mm, 5 μm, Waters).

## Results

### Purity of Ac-225 sources for radiolabeling

Before the optimization of the reaction conditions and QC techniques, the purity of the Ac-225 source was investigated. The Ac-225 stocks were diluted in 0.1 M HCl to a concentration of a 35 MBq/100µL. After radiolabelling, the RCY showed a decrease over time (RCY 99.9% in wk. 1, RCY 93.8% in wk. 4).

**Fig. 2:**
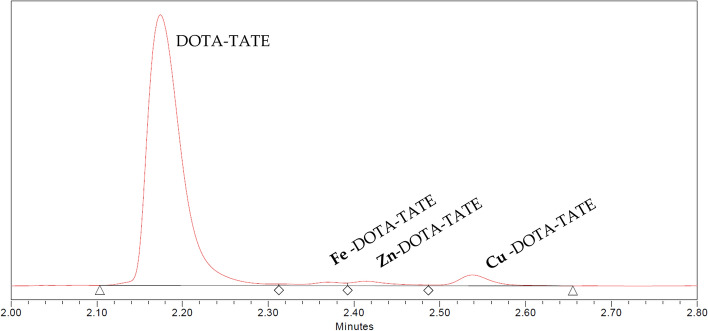
Example of UPLC-analysis of a Ac-225 stock (93.4% DOTA-TATE, 1.3% Fe-DOTA-TATE rf 2.38 min, 1.7% Zn-DOTA-TATE rf 2.42 min and 3.6% Cu-DOTA-TATE rf 2.55 min)

Trace metal quantification is performed at pH 4, as at a high pH, the metals are present in a hydroxide form, and do not complex with DOTA. Trace metals such as Fe^3+^, Zn^2+^ and Cu^2+^ form hydroxides at a high pH (Fe^3+^  ~ pH > 3 (Furcas et al. 2022), Zn^2+^  ~ pH > 7 (Zhang and Muhammed 2001) and Cu^2+^  ~ pH > 4 (Gabryel-Skrodzka et al. 2021)), while Ac-225 remains an ion at pH < 9 (Zielińska and Bilewicz 2004). The final metal concentration was corrected for the contribution of the matrix (Fig. 2).

**Fig. 3:**
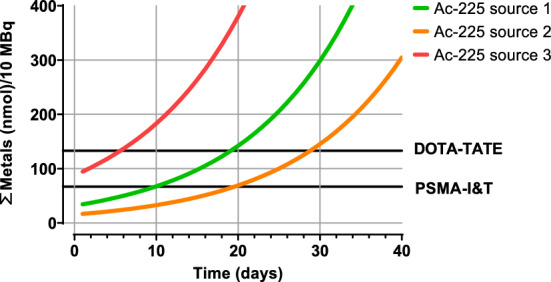
Amount of the trace metals/10 MBq (Σ(Fe, Zn and C), *y-axis*) in Ac-225 sources expressed in time (*x-axis*)

The metal content was measured and is expressed (ΣFe, Zn and Cu) in nmoles for 10 MBq Ac-225 (Fig. 3*)*. In time, a larger volume is required to maintain an activity of 10 MBq, leading to a relative increase in the total amount of added metals. Higher competition can be expected when the amount of metals exceeds the number of available chelators.

Based on the obtained results, different Ac-225 sources have a varying suitability for clinical applications. For example, based on previous studies, ± 130 nmol of DOTA-TATE (Blois et al. 2014) or ± 70 nmol of PSMA-I&T (Hooijman et al. 2022) is used for radiolabeling. For an application of 10 MBq [^225^Ac]Ac-PSMA-I&T, source 1 can be used for a maximum of 10 days, source 2 for up to 20 days, while for source 3 a low RCY can be expected from the start. For 10 MBq [^225^Ac]Ac-DOTA-TATE, source 1 can be used for up to 19 days, source 2 for more than 28 days, and source 3 for 5 days.

### Optimization of the RCY

**Fig. 4:**
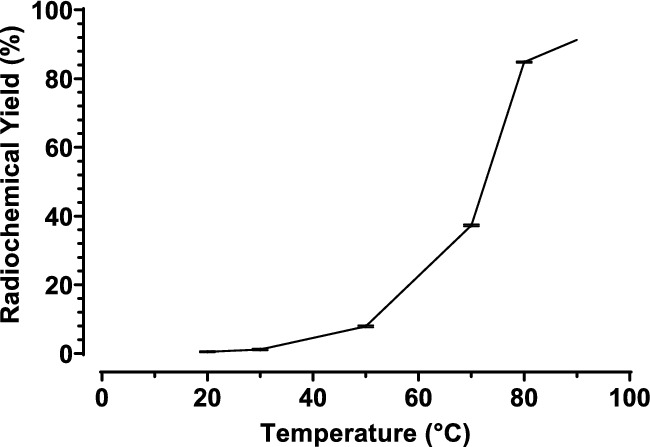
Standard labeling of [^225^Ac]Ac-DOTA-TATE at different temperatures (*n* = 3)

Radio-TLC using 0.5 M sodium citrate showed optimal peak shape and resolution. The heating time was investigated with radio-TLC, as described in Table 2. When the heating time (heating block) is increased from 10 to 20 min only a minor increase in RCY was obtained (87.2% ± 9.1 (*n* = 3) to 90.0% ± *1.5* (*n* = 3)), while standard deviation is decreased. Heating for 30 or 60 min shows no improvement of RCY (< 90%). When the temperature is increased, so does the RCY, whereby heating over 100 °C was not tested because of radiation safety (Fig. 4). After further optimization of pH, high RCY’s could be obtained, as described in Sect. ”Effect of pH”*.*. Additionally, a similar RCY was obtained when heating at 90 °C for 20 min in heating block (RCY 90.0% ± 1.5) in comparison with 5 min 95 °C in microwave (p > 0.05, RCY 90.8% ± 1.3).

### Development and optimization of HPLC-method for RCP determination

#### Optimization of HPLC-gradient

**Fig. 5:**
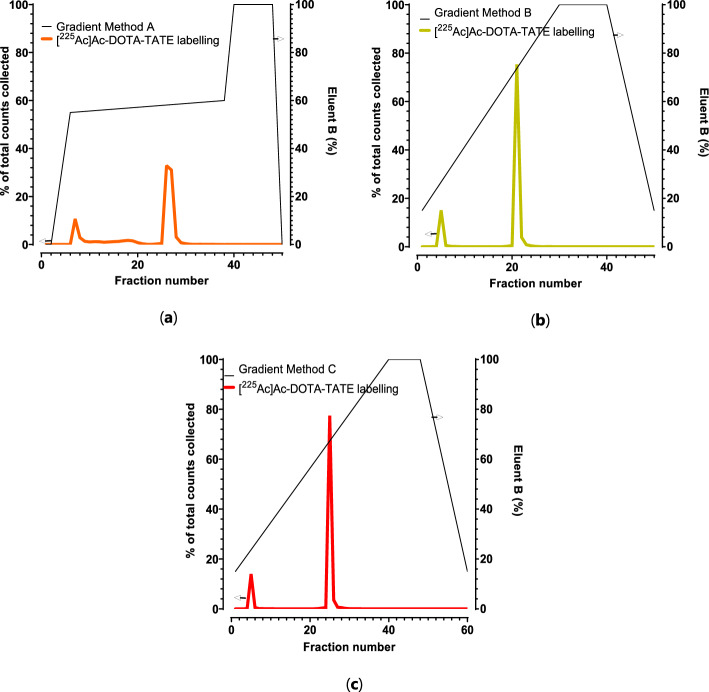
Comparison of the HPLC-methods, resulting in method A (**a**, RCP measurement 71.0%), method B (**b**, RCP measurement 83.1%) and method C (**c**, RCP measurement 84.8%)

The HPLC-method originally used for [^177^Lu]Lu-DOTA-TATE QC (Blois et al. 2012) was used as a starting point for the analysis of [^225^Ac]Ac-DOTA-TATE. To obtain the optimal separation between both unlabeled Ac-225/[^225^Ac]Ac-DTPA, radiolysis and [^225^Ac]Ac-DOTA-TATE, a labeling was injected in the HPLC-system, while varying the gradient. The method was adapted and focused on the separation of the first peak (dead volume) (Fig. 5).

Method A shows a low RCP value (71.0%) compared to method B (83.1%) and C (84.8%), suggesting a better separation of the impurities. Method A (Fig. 5a) showed a better separation of the radiolysis peaks (fraction 8–22) compared to method B (Fig. 5b) and method C (Fig. 5c). It is important to note that the RCYs from the radio-TLC and HPLC do not correspond, this is due to the overlapping peaks of Ac-225/[^225^Ac]Ac-DTPA and radiolysis.

#### Recovery and carry-over measurements

In our experience, the recovery showed to be a challenge for Ac-225 labeled radiopharmaceuticals (Aalbersberg et al. 2022). For Ac-225, due to radiation safety, only 10 kBq could be injected, this limitation adversely affected the recovery, which was initially observed to be < 60%. When diluting the HPLC-samples with a 25% EtOH (diluted in MilliQ) solution, the was recovery increased (Hooijman et al. 2022). Additionally, an RP C-18 column (LiChrospher® 100 RP-18 endcapped (4 × 250 mm, 5 µm) was investigated in comparison to the Symmetry C-18 column (5 μm 150 × 4.6 mm), resulting in a reduced stickyness and therefore improving the recovery. Changing the column resulted in a sufficient recovery (105.3 ± 3.4) and carry-over (0.5 ± 0.3). The 100 µL injection volume resulted in a carry-over of less than 1%. A dedicated HPLC-system showed to be essential, resulting in no interference of other radionuclides within the same energy window (Gillings et al. 2020).

#### Peak-resolution versus the amount of fractions

**Fig. 6:**
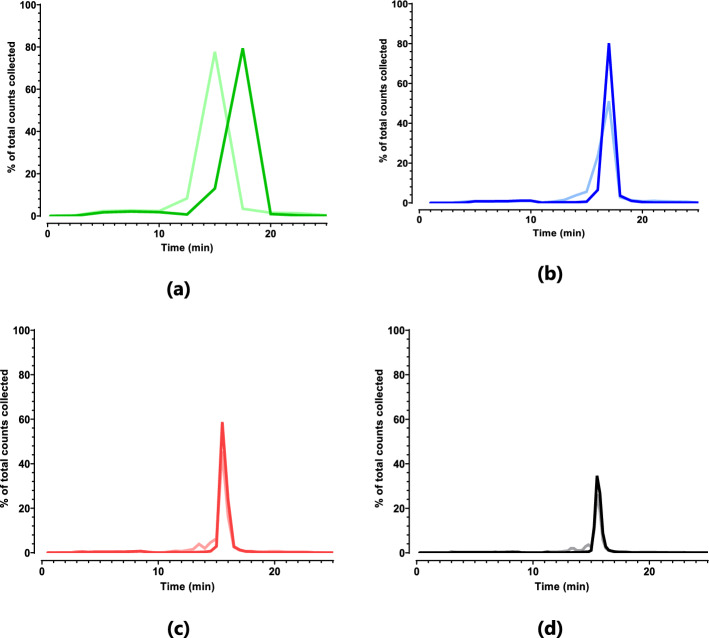
[^225^Ac]Ac-DOTA-TATE labeled in absence of quenchers to be able to visualize radiolysed radiopharmaceutical, t = 0 (*dark color*) and 48 h (*light color*) after radiolabeling. HPLC-injection (RCY > 98%) resulted in a different RCP measurements, of 10 fractions (**a**, 94.4%), 25 fractions (**b**, 92.5%), 50 fractions (**c**, 91.9%) and 100 (**d**, 91.3%), respectively

The peak resolution was monitored by changing the number of collected fractions after the injection of the [^225^Ac]Ac-DOTA-TATE sample (Fig. 6*)*, ranging from 10 fractions (Fig. 6a) to 100 fractions (Fig. 6d) obtained within the 30 min gradient. The peak separation and resolution consistently improved when the amount of collected fractions was increased, and the visualization of radiolysis becomes more detailed. At 10 fractions, there is insufficient resolution to separate impurities, this improved at 25 fractions, but it is only distinguishable at 50 fractions. Practically, 50 fractions is the most feasible within the given timeframe, handling and generation of waste. Additionally > 50 fractions results in activity measurements near the limit of quantification, which may result in an under- or overestimation of the RCP.

#### Radiolysis profiles

**Fig. 7:**
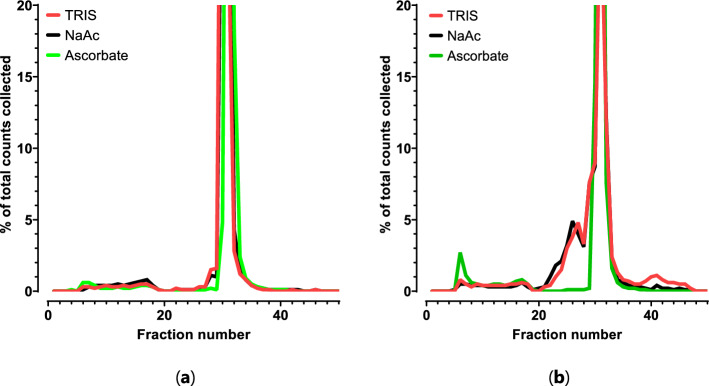
Indirect HPLC-analysis at t = 0 **a** and after 48 h **b** of [^225^Ac]Ac-DOTA-TATE labeling in presence of 15 mM TRIS, 15 mM sodium acetate or 35 mM ascorbate

The HPLC radiolysis profiles were investigated using different reaction conditions, whereby three different labeling (RCY > 98%) conditions showed differences in HPLC-profiles. The [^225^Ac]Ac-DOTA-TATE labeled in the presence of TRIS (15 mM, pH 8.5), sodium acetate (15 mM, pH 5.5) or ascorbate (35 mM, pH 5.5) (Table 2) showed different profiles at t = 0 (Fig. 7a) and after 48 h (Fig. 7b*),* and showed clear ingrowth of radiolysed radiopharmaceutical.

[^225^Ac]Ac-DOTA-TATE in the presence of TRIS or sodium acetate both showed identical HPLC-profiles, wereas in presence of ascorbate, an additional peak was formed (fraction 5–20).

### Effect of pH

**Fig. 8:**
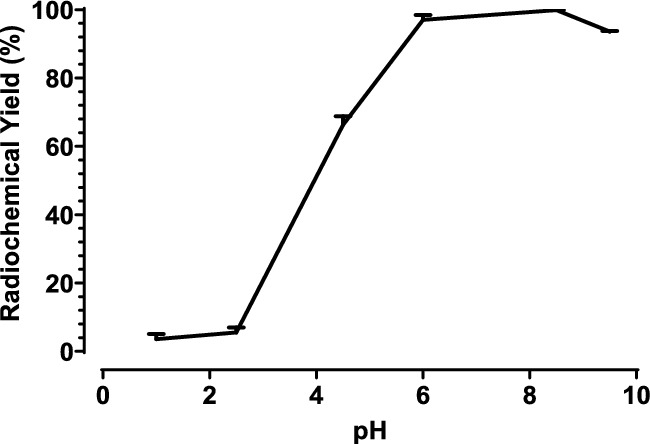
RCY by varying the pH

Initially, obtaining a RCY > 90% showed to be difficult. Initially, the labeling was performed at a low pH (pH < 4), however, since Ac-225 hydrolyzes at pH > 9 (Thiele and Wilson 2018), the labeling conditions at a higher pH resulted in a higher RCY (Fig. 8*)*. However, when the pH is over 9, a decrease in RCY was observed which is in concordance with Thiele et al. (Thiele and Wilson 2018).

Additionally, an excess of trace metals was added to simulate the metal impurities at different concentrations and pH. Consequently, labeling at a high pH shows a higher RCY, even when the excess of metals is further increased to 10x (metal over radiopharmaceutical) (Table 6).

**Table 6:** The RCY in the presence of trace metals (1 × or 10 × excess) at pH = 5.5 or pH = 8.5 (*n* = 2)

Excess of trace metals	pH	RCY (%)
1x	5.5	48.0 ± 34.7
1x	8.5	96.2 ± 3.0
10x	5.5	0.1 ± 0.1
10x	8.5	47.9 ± 35.6

### Influence of buffer

**Fig. 9:**
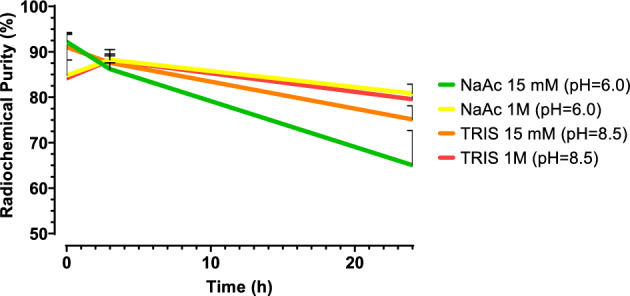
The RCP of [^225^Ac]Ac-DOTA-TATE labeled in presence of 15 mM/1 M sodium acetate or 15 mM/1 M TRIS up to 24 h after radiolabeling

Two buffers were tested at 2 different concentrations: 15 mM/1 M sodium-acetate (pH = 5.5) and 15 mM/1 M TRIS (pH = 8.5) (*n* = 2*, *Fig. 9) (Pretze et al. 2021; Reissig et al. 2021; Kratochwil et al. 2021). All labeling conditions resulted in a RCY > 95%. When a labeling was performed at a final concentration of 1 M, inhomogeneity was observed with both buffers. The stability of [^225^Ac]Ac-DOTA-TATE in presence of only sodium acetate or TRIS drops over time, which underlines the need of the addition of quenchers. 15 mM TRIS was selected for further investigation.

### Stability of [^225^Ac]Ac-DOTA-TATE after addition of different quenchers

**Fig. 10:**
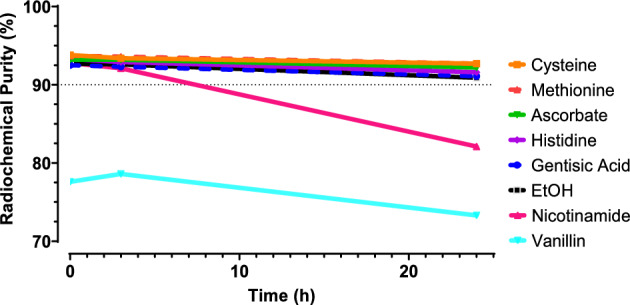
[^225^Ac]Ac-DOTA-TATE in the presence of cysteine, methionine, ascorbate, histidine, gentisic acid, nicotinamide, vanillin (35 mM) and 10% v/v EtOH up to 24 h after radiolabeling,

Quenchers are required to be able to have a high RCP and longer stability in time. Therefore, the stability of [^225^Ac]Ac-DOTA-TATE was monitored after addition of different quenchers (Blois et al. 2014). For the initial comparison, standardized labeling conditions were used as described in Tables 2 and 3. The quenchers cysteine, methionine, ascorbate, histidine, gentisic acid and EtOH showed an RCY > 99%, and a RCP of > 90% up to 24 h (Fig. 10*)*. However, nicotinamide and vanillin show a limited effect on the stability and additionally resulted in a lower RCY (nicotinamine 98.4%/vanillin 99.9%) and RCP at 24 h (nicotinamide 82.1%/vanillin 73.0%).

### Labeling volume

The volume of the radiolabeling might also influence RCY and/or stability. While an increased volume alters the kinetics of the radiolabeling, it may also reduce the optimal concentration of quencher(s), leading to an increase in radiolysis of the radiopharmaceutical. For the described experiments, the quencher concentration is kept constant while varying volume. The labeling volume up to 1 mL showed acceptable outcomes (RCP > 94% up to 24 h) (Table 3*)*. It should be noted that radiolabeling in 0.1 mL shows the best stability (RCP > 97% up to 24 h). Concerning the volume, the smaller the volume, the higher the RCP can be maintained in time. Increasing the volume to 10 mL before heating resulted in only limited yields (RCY/RCP < 90%).

### Molar activity

The molar activity experiments were performed based on clinical conditions (Table 3). TRIS-based labeling conditions (pH = 8.5) resulted in a molar activity up to 270 kBq/nmol (RCP 90.8%) and radiolabeling using ascorbate (pH = 5.5) resulted in a molar activity up to > 540 kBq/nmol (RCP 91.8%).

### Storage conditions

Storage of [^225^Ac]Ac-DOTA-TATE on either dry ice or freezing by -20 °C was investigated. All conditions had a RCY of > 95%. Storage within a cold environment shows minor improvements in stability (RCP): + 1.5% for dry-ice after 24 h and + 2.7% increase when stored at − 20 °C after 48 h.

## Discussion

Currently, multiple Ac-225 sources are available, and show to be useable up to 4 weeks after dilution. However, this is dependent on the metal content and pH that is used during labeling. This lifespan allows flexibility in experimental timelines and reduces resource wastage. However, when labelling at higher pH, the buffer capacity of the used TRIS buffer (pH = 8.5) is relatively low. The final labeling volume does influences the RCY, therefore the Ac-225 can only be used up to 2 weeks. Other buffers at a high pH might have better buffering capacity. Buffers such as HEPES (pH = 6.8–7.2) or ammonium acetate (pH = 7.0) used for Zr-89 radiolabeling might be of benefit and have to be futher investigated (Wuensche et al. 2024).

The ingrowth of daughter nuclides is often overestimated, as they are typically present in the low picomolar range, whereas the present concentrations of Cu, Zn, and Fe, are in the nanomolar range (Hooijman et al. 2024). For the determination of metals such as Fe, Zn, and Cu, it is essential to perform labelling at a low pH (pH = 3), as the formation of hydroxide species at higher pH levels may result in an underestimation of their concentrations. This hypothesis is further supported by addition of an excess of metals showing a better performance at a high pH (Sect. ”Effect of pH”). Depending on the intended clinical application and the molar activity that is used for radiolabeling, the amount of metal-ions relative to the amount of MBq (Ac-225) may pose a challenge and may influence the labeling kinetics. When lower RCY’s are obtained, purification of the final product could be an option, however, this process will complicate the procedure. Working metal free (cleaning with Chelex), may improve the labeling conditions and might result in higher RCY so that futher purification is not longer required (Rayner and Suzuki 1995). Furthermore, the efficiency of metal incorporation into the DOTA-chelator is driven by the affinity for metals-ions with a 2/3^+^ charge. Other chelators such as Macropa (Kadassery et al. 2022), Crown (Yang et al. 2020) and H4py4pa (Li et al. 2021) might posses a higher specificity, but require individual optimization (Breeman et al. 2014).

Firstly, the labeling conditions in regards to RCY are highly dependent on heating time and temperature. A microwave showed to be more practically suitable for clinically scaled activity radiolabelings due to the guaranteed closed system at high pressures (up to 30 bar) and subsequent better heat transfer (Hooijman et al. 2021).

Thereafter, to be able to optimize the RCP, it is important to note that HPLC-gradient optimalization (Fig. 5) and resolution (Fig. 6) were of high importance. As one of the main challenges in radiopharmaceutical development is the detection of radiolysed radiopharmaceutical. Therefore, the HPLC-gradients were adapted to enhance the separation of the radiolysis peak. However, no notable improvements were observed. But the use of a phosphate buffer may provide a more base-to-base separation of the impurities (Kraihammer et al. 2023).

Optimal visualization of radiolysis requires a sufficient amount of fractions. In this example, when using less than 50 fractions, quantification of the radiolysis degradation products was not feasible and this resulted in an overestimation of the RCP. On the contrary, when increasing the amount of fractions to > 50, the measurements are more towards the LOD of the detection methods, which may increase the error of the measurements (Saldarriaga Vargas et al. 2020).

To verify the detection of radiolysis in the selected methods as described (Fig. 7) (Pretze et al. 2021; Kratochwil et al. 2021; Kratochwil et al. 2020), it is notable that different profiles can be observed. A buffer is included to stabilize the pH and influences the H^+^/OH^−^ ion concentration and therefore might change the reactive radical species that are formed during radiolabeling (Larenkov, et al. 2023). Consequently, different formed reactive radical species are developed and are likely dependent on the isotope and might have a different impact on the used biological vector, buffer and/or quencher. Furthermore, quenchers can neutralize the reactive species (Chen et al. 2008), but may also cause an interaction with the compound, which may be observed in the form of additional peaks which has been observed in the presence of vanillin (*data not shown*). Due to the low mass of precursor, LC–MS methods will be of no use and the exact composition remains therefore unknown. Furthermore, understanding the biodistribution of the degradation products in an in vivo setting, will offer valuable insights into the toxicity and patient safety (Feuerecker et al. 2021; Raitanen et al. 2024; Kruijff et al. 2019).

In conclusion, the optimal RCY and RCP in this setting was obtained with a heating temperature of > 80 °C, a pH of > 8.5 (15 mM TRIS), with the quencher cysteine, methionine, ascorbate, histidine or genticic acid, in a small volume (< 1 mL). Additionally, as demonstrated by Ac-225 labeling with DOTA(-TATE), the obtained results are not straightforward, emphasizing the need for customized optimization of the analytical methods for each Ac-225 labeled radiopharmaceutical to ensure optimal performance and safety in a clinical setting.

## Conclusion

The purity of Ac-225 labeled DOTA-radiopharmaceuticals is dependent on the pH and composition of the reaction mixture. This research has shown that labeling Ac-225 at higher pH reduces the influence of metal impurities. Optimized radiolabeling conditions, accurate measurements and improved quality control methods are essential for achieving an optimal measurement of the RCY and RCP/stability. Optimization of these parameters is strongly recommended to ensure effective and safe patient treatment.

## Data Availability

The datasets used and/or analyzed during the current study are available from the corresponding author on reasonable request.
